# No differences in hemostatic and endothelial activations between haploidentical and matched-donor hematopoietic stem cell transplantation in thalassemia disease

**DOI:** 10.1186/s12959-020-00232-z

**Published:** 2020-09-04

**Authors:** Surapong Lertthammakiat, Peerasit Sitthirat, Usanarat Anurathapan, Duantida Songdej, Samart Pakakasama, Ampaiwan Chuansumrit, Nattaphat Putawornsub, Sawitt Sirasittikarn, Sataporn Wantanawijarn, Praguywan Kadegasem, Suradej Hongeng, Nongnuch Sirachainan

**Affiliations:** 1 Department of Pediatrics, Faculty of Medicine Ramathibodi Hospital, Mahidol University, 270 Rama VI Road, Ratchathewi District, Bangkok, Thailand; 2 Chakri Naruebodindr Medical Institute, Faculty of Medicine Ramathibodi Hospital, Mahidol University, Samut Prakan, Thailand; 3 Department of Biology, Mahidol Wittayanusorn School, Nakhon Pathom, Thailand

**Keywords:** Endothelial activation, Hemostasis, Thalassemia, Haploidentical hematopoietic stem cell transplantation

## Abstract

Hemostatic changes and endothelial activations have been recognized in β-thalassemic patients after matched-donor hematopoietic stem cell transplantation (HSCT) but there are limited studies for haploidentical HSCT. This report demonstrates that the levels of hemostatic and endothelial markers, including thrombin antithrombin complex, prothrombin fragment, D-dimer, von Willebrand factor antigen and thrombomodulin levels, were not significantly different between haploidentical and matched-donor HSCT patients.

## Key points

1. HSCT-induced hemostatic changes and endothelial activations
2. Haploidentical and matched-donor HSCT had similar hemostatic changes and endothelial activations
3. Evidence of hemostatic changes and endothelial activations presented during the first year after HSCT

## Background

β-thalassemia disease is caused by reduced or absent β-globin chain synthesis. Most patients with these conditions have moderate to severe anemia, requiring regular red blood cell transfusions [1–4]. The curative treatment for severe thalassemia disease is a matched-donor hematopoietic stem cell transplantation (HSCT). However, the chance of having a matched-donor HSCT is less than 50%. At present, haploidentical HSCT has been increasingly used to treat various diseases, including malignancy and hemoglobinopathy. Although haploidentical HSCT is expected to have more complications after HSCT, protocols to reduce complications and graft failure have been developed, such as CD34 selection with reduced T cells as a stem cell source. In previous studies, in order to reduce complications, patients who underwent haploidentical HSCT received two cycles of pre-transplantation immunosuppression before a conditioning regimen of fludarabine and busulfan [5–7]. The same studies had a conditioning regimen of busulfan and cyclophosphamide for matched-donor HSCT. To address graft versus host disease (GvHD) prophylaxis, post-transplantation treatment with cyclophosphamide was added to the standard immunosuppressive regimen of calcineurin inhibitors and mycophenolate mofetil or methotrexate. As a result, patient outcomes were comparable to matched-donor HSCT patients [5, 8–11].

Changes in hemostasis have been recognized during and post HSCT (95% of patients received matched-donor HSCT), due to high dose chemotherapy, radiation, inflammation, immune response, and GvHD, resulting in endothelial activation and increased tissue factor. Evidence of these changes can be seen in increased tumor necrosis factor-α (TNF-α), von Willebrand factor antigen (vWF:Ag), thrombin-antithrombin complex (TAT), prothrombin fragment 1 + 2 (F1.2) and D-dimer, and decreased anticoagulation proteins [12–15]. As a result of these changes, increased risks of bleeding and thrombosis were observed in HSCT patients. In thalassemia, the activation of coagulation has been reported before HSCT, mainly from abnormal red blood cell surfaces, endothelial and platelet activations [13, 16–18]. The delayed recovery of the hemostatic system after HSCT in thalassemia has been reported, especially in patients with matched-donor HSCT, however, to our knowledge, there is no report regarding hemostatic changes in thalassemia patients who received haploidentical HSCT compared with matched-donor HSCT. Therefore, the study aims to compare hemostasis and endothelial activation in thalassemia post-haploidentical HSCT with matched-donor HSCT.

## Materials and methods

### Subject enrollment and laboratory testing

This study was a cross-sectional study performed at the Department of Pediatrics, Faculty of Medicine Ramathibodi Hospital from November 2014 to October 2015. It was approved by the Committee on Human Rights Related to Research involving human subjects. This study enrolled four groups of individuals: 1) severe β-thalassemia patients who underwent haploidentical HSCT, 2) severe β-thalassemia patients who underwent matched-donor HSCT, 3) thalassemic patients receiving regular red blood cell transfusion (Thal-RT), and 4) healthy subjects who had normal Hb and red blood cell indices (NC). Groups 3 and 4 were enrolled as control groups.

Blood from all subjects was tested for vWF:Ag (Dako, Glostrup, Denmark), thrombomodulin (Abcam®, Cambridge, UK), TAT (Enzygnost® TAT micro, Dade Behring AG, Marburg, Germany), F1.2 (Enzygnost® F 1 + 2, Dade Behring AG, Marburg, Germany), and TNF-α (R&D systems®, Minneapolis, USA). These levels were measured using the ELISA technique, and D-dimer (Innovance® D-dimer, Siemens AG, Munich, Germany) was measured in an automated quantitative latex-enhanced immunoturbidimetric assay. Univariate analysis, Bonferroni, and Mann-Whitney U tests were used to compare the nonparametric data among groups with confounding factors.

### Pharmacological pre-transplant immunosuppression

Pharmacological pre-transplant immunosuppression consisted of fludarabine 40 mg/m^2^/day together with dexamethasone 25 mg/m^2^/day intravenously for 5 days and was given to all patients with haploidentical HSCT and matched-donor HSCT with ages ≥7 years. This protocol was used inaccordance with a previous study [7]. The protocol was administered in two courses: the first on days HSCT − 68 to − 64, and the second on days HSCT − 40 to − 36. The pharmacological pre-transplant immunosuppression used was aimed to suppress recipient T-cell function and facilitate engraftment. Reduced-toxicity conditioning was also done and comprised of intravenous fludarabine 35 mg/m^2^/day for 6 days, busulfan 130 mg/m^2^/day for 4 days and anti-thymocyte globulin 1.5 mg/kg/day for 3 days. For the GvHD prophylaxis regimen, cyclophosphamide 50 mg/kg/day was administered intravenously on days HSCT + 3 to + 4. A calcineurin inhibitor, either tacrolimus or cyclosporine, and mycophenolate were given as prophylaxis for GvHD.

### Statistical analysis

SPSS 18.0 (SPSS Inc., Chicago, IL, USA) was used to compute all statistical analyses. The nonparametric data among groups was compared with a Kruskal Wallis H test, and categorical data was compared with a Chi-Square test. Univariate analysis, Bonferroni, and Mann-Whitney U tests were used to compare the nonparametric data among groups with confounding factors. The correlation of variables was assessed by a Pearson correlation coefficient and Mann-Whitney U test. A *p*-value of < 0.05, was statistically significant.

## Results

A total of 23 Thal-HSCT, including 14 patients with haploidentical HSCT and 9 patients with matched-donor HSCT, were enrolled. The median (range) time at enrollment since HSCT was 54 (15–331) days and 87 (35–296) days for haploidentical and match-related donor HSCT, respectively. In addition, 28 β-thalassemic patients who did not receive HSCT (Thal-RT) and 20 NC were included as comparative groups. The age of the patients in the Thal-HSCT [10.2 (92.7–20.2) years] group was not significantly different when compared to the Thal-RT group [11.8 (1.9–16.8) years]. There was no difference in the gender ratios between all groups. Almost all the patients in both Thal-HSCT (87%) and Thal-RT (79%) groups were diagnosed with HbE/β thalassemia. The remaining patients were diagnosed with β-thalassemia major. Patients in the Thal-RT group had been receiving regular red blood cell transfusions with a median pre-transfusion hemoglobin level of 9.0 g/dL (range: 7.0–11.0). Their median level of serum ferritin was 2050 ng/ml (range: 268–7790). When comparing between haploidentical HSCT and matched-donor HSCT groups, no differences in age, ferritin level and hepatomegaly were found. The prevalence of GvHD was higher in the haploidentical HSCT group when compared to the matched-donor HSCT group (50% vs 33%, Table 1). Infection occurred in 70% of haploidentical HSCT patients and 33% in matched-donor HSCT patients. In haploidentical HSCT, 7 patients developed viral infection; 3 with cytomegalovirus, 1 with BK virus, 1 with adenovirus, 2 with BK and CMV virus, and 3 patients developed bacterial infections (1 with *Acinetobacter pitti* septicemia, 1 with *Pneumocystis carinii* pneumonia and 1 with folliculitis). In the matched-donor HSCT group, 3 patients developed viral infection (2 with cytomegalovirus and 1 with BK virus). One patient had mixed chimerism (76.5%) at the time of the study and later developed graft loss and received a second HSCT.

**Table 1:** Demographic data for Thal-HSCT patients by donor group: haploidentical and matched-donor

	Type of donor		***P***-value
	Haploidentical ***n*** = 14	Matched-donor ***n*** = 9	
**Type of thalassemia,** n (%)			0.295^a^
HbE/β	13 (93)	7 (78)	
β major	1 (7)	2 (22)	
**Age at HSCT in years,** median (range)	10.6 (2.7–18.9)	8.2 (3.8–20.2)	0.926^b^
**Day after HSCT in days,** median (range)	54 (15–331)	87 (35–296)	0.374^b^
**Ferritin in ng/ml,** median (IQR)	2972.6	3512.7	0.913^b^
**Hepatomegaly,** n (%)	5 (56)	6 (75)	0.147^a^
**Classification,** n (%)			
Age < 7 years, no hepatomegaly	0 (0)	0 (0)	
Age > 7 years, no hepatomegaly	4 (44)	2 (25)	
Age < 7 years, hepatomegaly	0 (0)	2 (25)	
Age > 7 years, hepatomegaly	5 (56)	4 (50)	
**Conditioning regimen,** n (%)			
Bu + Cy		2 (22)	
ATG + Flu + Bu	14 (100)	7 (78)	
**Chimerism,** n (%)			0.412^a^
Complete donor	13 (93)	9 (100)	
Mixed	1* (7)		
**Veno-occlusive disease,** n (%)	2 (14)	3 (33)	0.280^a^
**Graft-versus-host disease,** n (%)			
Acute GvHD			
Grade I/II	7 (50)	2 (22)	0.171^a^
Grade III/IV	0 (0)	1 (11)	
Chronic GvHD	1 (7)	0 (0)	0.412^a^
**Infection,** n (%)			
Bacterial	3 (21)	0 (0)	0.136^a^
Viral	7 (50)	3 (33)	0.431^a^

*IQR* interquartile range, *GvHD* graft versus host disease

Conditioning regimen: *ATG* Thymoglobulin, *Bu* busulfan, *Cy* Cyclophosphamide, *Flu* fludarabine

*Donor chimerism 76.5%

*All patients in the haploidentical HSCT group received pre-transplant immunosuppression (PTIS), 7 patients who were > 7 years old in the matched-donor HSCT group received PTIS (ref. 7) PTIS consisted of fludarabine 40 mg/m^2^/day and dexamethasone 25 mg/m^2^/day

Statistical analysis performed using: ^a^ Chi-Square, ^b^ Bonferroni

The results demonstrated that vWF:Ag and thrombomodulin levels in the Thal-RT and Thal-HSCT groups were significantly higher than those in the NC group. However, there was no significant difference between haploidentical and matched-donor HSCT groups for both vWF:Ag and thrombomodulin levels. When comparing TAT, F1.2, and D-dimer levels between haploidentical and matched-donor HSCT, there were no significant differences seen. TNF-α levels were significantly higher in Thal-RT and matched-donor HSCT groups when compared to that in NC group. When comparing TNF-α levels between haploidentical and matched-donor HSCT patients, there was also no significant difference found although the prevalence of GvHD was higher in the haploidentical group when compared to the matched-donor HSCT group (50% vs 33%, Table 1 and 2).

**Table 2:** Laboratory results in normal control (NC), beta-thalassemic patients receiving regular blood transfusion (Thal-RT) and post-hematopoietic stem cell transplantation beta-thalassemic patients (Thal-HSCT)

Parameter	NC *n* = 20	Thal-RT *n* = 28	Thal-HSCT Classified by type of donor	
			Haploidentical *n* = 14	Matched-donor *n* = 9
VWF:Ag (%)	80.8 (67.5–108.1)	131.3^*^ (100.3–151.5)	149.7^*^ (127.9–162.1)	139.4^*^ (113.2–169.9)
TNF-α (pg/ml)	0.1 (0.0–2.2)	4.4^*^ (3.1–5.3)	3.2 (2.8–4.4)	4.7^*^ (3.8–5.2)
TM (ng/ml)	2.7 (2.3–3.4)	ND	3.8 (2.5–4.2)	4.0 (3.5–5.5)
F1.2 (pmol/l)	125.5 (109.8–150.9)	404.1 (195.7–1048.0)	227.6^#^ (173.2–281.2)	213.8 (190.1–271.5)
TAT (μg/l)	2.6 (2.4–3.0)	4.7 (2.0–7.0)	3.0 (2.6–4.0)	4.7 (3.7–6.3)
D-dimer (ng/ml)	224.0 (118.0–346.5)	287.2 (181.6–453.2)	254.0 (207.0–429.0)	410.0 (335.0–555.0)

Abbreviations: *vWF:Ag* von Willebrand factor antigen; *TNF-α* tumor necrosis factor alpha; *TM* thrombomodulin; *F1.2* prothrombin fragment 1 + 2; *TAT* thrombin-antithrombin complex; *ND* no data

Data expressed as median (interquartile range)

*, significant compared with NC, *p*-value < 0.05

#, significant compared with Thal-RT, *p*-value < 0.05

tested by Bonferroni

## Discussion

The activation of hemostasis during and after HSCT has been shown in a previous report, but most of the patients received matched-donor HSCT, with only a few patients (5%) receiving haploidentical HSCT [13]. Therefore, the results of activation of hemostasis in the previous report were mostly from matched-donor HSCT. The present report included 23 patients who received HSCT, with 60.8% of patients received haploidentical HSCT. The HLA disparity, present in haploidentical HSCT, has been reported to increase the risk of HSCT-related complications such as GvHD and graft loss, resulting in increased inflammation, hemostatic changes, and endothelial activations. This report, therefore, compared these elements between haploidentical and matched-donor HSCT patients during the first year after HSCT. It is during this time when there is the most hemostatic disturbance. Thal-RT and NC subjects were included as comparison groups. Although, the age in the NC group was higher than in the Thal-RT and Thal-HSCT groups, a Univariate analysis was used to adjust all affected parameters, including age.

Markers for hemostatic change and endothelial activation, including TAT, F1.2, D-dimer, vWF:Ag and thrombomodulin, were used to represent the hemostasic change and endothelial activation in this report. From the results, the hemostatic changes and endothelial activation were not significantly different between haploidentical and matched-donor HSCT groups. Although the number of patients who received HSCT in this study was relatively low (*n* = 23), to our knowledge, it is the first report to suggest that following the two-phase treatment protocol (outlined above) with haploidentical HSCT patients, not only improves patient outcome, but also results in comparable hemostatic change and endothelial activations when compared to matched-donor HSCT patients following the same protocol. A larger cohort is required to confirm these results.

TNF-α, one of the cytokines that elevates during HSCT, was also studied, in order to determine the inflammatory response after HSCT. In the present report, TNF-α levels were elevated in Thal-RT and both HSCT groups when compared to the NC group. In a previous study, high TNF-α level had been demonstrated in β-thalassemia patients, which were a result of activation of macrophages from iron overload [19]. Another study also showed high TNF-α level during HSCT because of the conditioning regimen, inflammation and GvHD [20]. In this present study, the TNF-α levels were similar between haploidentical and matched-donor HSCT groups, although the prevalence of GvHD in the haploidentical HSCT group was higher than that in the matched-donor HSCT group. Only GvHD grade I-II occurred in haploidentical HSCT.

This preliminary study provided evidence of the possibility of attaining similar hemostatic changes and endothelial activations in haploidentical and matched-donor HSCT patients with thalassemia disease. For thalassemia patients who undergo haploidentical HSCT, a reduced intensity conditioning regimen combined with post-transplantation cyclophosphamide prophylaxis, may be effective in controlling hemostatic changes and endothelial activations. Further study is required to confirm these findings.

## Abbreviations

F1.2: Prothrombin fragment 1 + 2
GvHD: Graft versus host disease
HSCT: Hematopoietic stem cell transplantation
TAT: Thrombin-antithrombin complex
TNF-α: Tumor necrosis factor-α
vWF:Ag: Von Willebrand factor antigen
